# The Dirichlet Dual Response Model: An Item Response Model for Continuous Bounded Interval Responses

**DOI:** 10.1007/s11336-023-09924-7

**Published:** 2023-07-20

**Authors:** Matthias Kloft, Raphael Hartmann, Andreas Voss, Daniel W. Heck

**Affiliations:** 1 Department of Psychological Methods, University of Marburg, Gutenbergstr. 18, 35032 Marburg, Germany; 2 Heidelberg University, Heidelberg, Germany

**Keywords:** response formats, dual range slider, item response theory, interval responses, continuous bounded responses, variability in behavior, uncertainty

## Abstract

Standard response formats such as rating or visual analogue scales require respondents to condense distributions of latent states or behaviors into a single value. Whereas this is suitable to measure central tendency, it neglects the variance of distributions. As a remedy, variability may be measured using interval-response formats, more specifically the dual-range slider (RS2). Given the lack of an appropriate item response model for the RS2, we develop the Dirichlet dual response model (DDRM), an extension of the beta response model (BRM; Noel & Dauvier in Appl Psychol Meas, 31:47–73, 2007). We evaluate the DDRM’s performance by assessing parameter recovery in a simulation study. Results indicate overall good parameter recovery, although parameters concerning interval width (which reflect variability in behavior or states) perform worse than parameters concerning central tendency. We also test the model empirically by jointly fitting the BRM and the DDRM to single-range slider (RS1) and RS2 responses for two Extraversion scales. While the DDRM has an acceptable fit, it shows some misfit regarding the RS2 interval widths. Nonetheless, the model indicates substantial differences between respondents concerning variability in behavior. High correlations between person parameters of the BRM and DDRM suggest convergent validity between the RS1 and the RS2 interval location. Both the simulation and the empirical study demonstrate that the latent parameter space of the DDRM addresses an important issue of the RS2 response format, namely, the scale-inherent interdependence of interval location and interval width (i.e., intervals at the boundaries are necessarily smaller).

## Introduction

Personality psychology has a decades-long tradition of using response scales to measure traits (Likert, 1932; Thurstone, 1929). In standard personality inventories, respondents answer questions or statements by condensing a wide range of attitudes, experiences, and behaviors into a single response value. In contrast to standard practice, whole trait theory (Fleeson & Jayawickreme, 2015) conceptualizes personality traits as density distributions of states. Fleeson (2001) showed in a series of experience-sampling studies that not only the central tendencies of these state distributions, but also their variances, are stable person characteristics. Consequently, a single response to an item can be viewed as an aggregate summary reflecting the central tendency of a distribution of states within a respondent. Usually, however, the variance of internal distributions is neither measured nor modeled. This can be problematic because two respondents having personality state distributions of different variability could end up choosing the same response value on the response scale, which might in turn lead researchers to assume equivalence with respect to the latent construct, while in reality the two individuals differ with respect to their experiences.

As a solution, it might be possible to measure the variability of internal distributions of states or behaviors using an interval-response format. For each question or statement, respondents set a lower and an upper bound to indicate a range of values that best represent their attitudes, behaviors, or experiences. Such an approach can lead to different statistical conclusions compared to using Likert-type scales (Lubiano et al., 2016).

Ellerby et al. (2022) showed that interval responses are a promising approach for psychometric measurement in general. Using an interval-response format, respondents were able to adequately indicate both objective and subjective variance. The authors also describe two types of interval responses that represent qualitatively different sets of values (for a more in depth discussion, see Couso & Dubois, 2014). First, disjunctive sets include only one value that is considered to be the normatively correct answer. Response intervals that represent disjunctive sets allow respondents to express uncertainty about the correct answer, for instance, when answering general-knowledge questions (e.g., “What is the height of the Eiffel tower?”). Second, a response interval may represent a conjunctive set which consists of values that are all true or valid answers. For instance, in a personality questionnaire, a respondent may provide a range of plausible values for a question or statement, which might reflect their variability in behaviors or flexibility in reacting to situational demands. Response intervals representing conjunctive sets are thus at the focus of the present article.

Based on the findings of Ellerby et al. (2022), we assume that the location of a response interval still reflects the central tendency of the underlying latent trait equivalently as for a single-response format. Further, we assume that the width of a response interval is an indicator of trait variability that reflects the variance of the distribution of states (Ellerby et al., 2022). However, the interpretation of the interval width will change depending on the specific use case for the interval response format. We therefore use the more neutral term “expansion dimension” to refer to the corresponding latent dimension, which is the hypothesized variability of latent states in our motivating example (i.e., whole trait theory). The intended interpretation of the expansion dimension for a given application needs to be treated with caution and should be validated, for instance, using experimental studies. To facilitate empirical tests of the assumptions and interpretations mentioned above, we develop a psychometric model for measuring trait variability via interval responses.

Given that we aim at modeling the variability of latent traits, our approach is an alternative to so-called *variable*-$\theta$ *models* (Ferrando, 2011, 2014). In the variable-$\theta$ approach, variability is conceptualized at the respondent level. A response to an item is assumed to be generated by the current, momentary trait level of the respondent, which fluctuates around a stable, person-specific mean of the trait. The amount of variability in the latent trait is modeled by a person-specific variance parameter, which can be interpreted as the respondent’s reliability across the whole set of items. In contrast, our approach directly infers the variability of behaviors and states from the responses at the *item level* (operationalized by the width of a response interval).

**Fig. 1:**
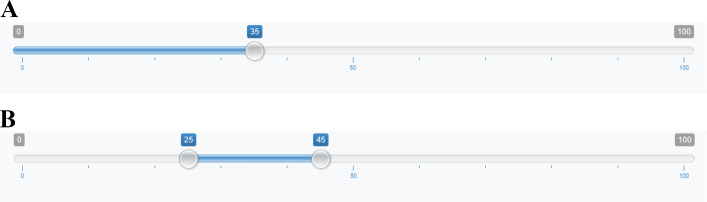
Single-range slider (Panel A) and dual-range slider (Panel B). *Note.* The sliders were created with the Ion.RangeSlider java plugin (Ineshin, 2021).

One convenient implementation of an interval-response format is the dual-range slider (RS2; see Appendix A for a list of abbreviations) shown in Fig. 1B. Using a web browser or any experimental software, respondents have to adjust two slider handles in order to obtain a response interval of a certain location and width. Thus, the response forms a bounded segment on a continuous response scale. Compared to categorical answers, the continuous scale of the RS2 provides a higher resolution of response options, which in turn allows respondents to give finer-grained answers and allows for interval-scale measurement (Reips & Funke, 2008). This is especially important in the present application where the mutual constraint of lower and upper bounds naturally decreases the number of possible response values for either one of the sliders. Another benefit of relying on a continuous scale is that the corresponding item response models are usually more parsimonious than those for categorical data because they do not require multiple category threshold parameters (Noel & Dauvier, 2007).

### Item Response Theory Models for Continuous Bounded Responses

Computerized tests have made it easy to implement continuous response scales for data collection, usually via single-range sliders (RS1) as shown in Fig. 1A. The idea is not novel though. Outside of the digital world, continuous scales have been known for a long time as graphic rating scales or visual analog scales. According to Yeung and Wong (2019), a graphic rating method was first mentioned by Hayes and Patterson (1921). Continuous scales have since been used regularly to measure various constructs such as the strength of pain in clinical settings (e.g., Bijur et al., 2001). From a modeling perspective, several item response theory (IRT) models have been proposed for the evaluation and scoring of continuous scales such as the RS1 (Ferrando, 2001; Mellenbergh, 1994; Müller, 1987; Noel & Dauvier, 2007; Samejima, 1973; Deonovic et al., 2020). However, to the best of our knowledge, IRT models for continuous interval responses have not yet been proposed. The present article addresses this gap by developing such a model.

Bounded responses often have a skewed distribution (Verkuilen & Smithson, 2012), which renders the normal distribution an inappropriate choice for modeling. A specific challenge thus concerns the mapping of the bounded space of the manifest response scale to an unbounded latent parameter space. The continuous response model (Samejima, 1973) addresses this issue with a transformation approach. After applying a logit transformation to the responses, latent values are assumed to be normally distributed (Wang & Zeng, 1998). In contrast, Müller (1987) and Ferrando (2001) used a truncation approach assuming that unbounded latent responses are normally distributed. If latent responses fall outside the range of the manifest response scale, they are simply truncated and redistributed during the response process.

Other models for bounded responses completely omit the assumption of an underlying normal distribution. The approach by Deonovic et al. (2020) divides the continuous response into conditionally independent binary variables that each follow a Rasch model (Rasch, 1993). Moreover, Noel and Dauvier (2007) proposed a response mechanism in terms of agreement and disagreement that is parameterized using a beta distribution. In addition to its ability to account for heavily skewed distributions, the beta distribution offers the advantage that it directly generalizes to the Dirichlet distribution if more than one response is observed on the bounded scale. Thus, the beta response model (BRM; Noel & Dauvier, 2007) is an ideal candidate for a model extension that applies to interval responses. However, when providing two values on a shared scale (i.e., lower and upper bound of an interval response), the inherent constraints on possible responses become even more severe. The two bounds of a response interval are bounded by the lower and upper end of the scale, and additionally, the lower bound necessarily has to be below the upper bound. As a remedy, the Dirichlet distribution offers the benefit of taking the scale-inherent constraints and interdependencies into account. Hence, we decided to rely on the BRM as a basis for developing a model that accommodates interval responses via a Dirichlet distribution.

### Aims

The first aim of the present article is to propose a novel IRT model, the Dirichlet dual response model (DDRM), which accounts for interval responses on a continuous bounded scale. For this purpose, we evaluate parameter recovery in a simulation study. Moreover, we assess the model’s fit to data in an empirical example for an Extraversion questionnaire based on posterior predictive checks and leave-one-out cross-validation.

The second aim concerns the validation of the person parameters of the proposed IRT model. We assume that the locations of the response intervals of the RS2 correspond to the central tendency of a latent trait. To test this assumption, we assess the convergent validity of the model’s location parameters by comparing the corresponding estimates to those obtained by fitting the BRM to RS1 responses. We expect a high correlation (i.e., $r >.70$, comparable to reliability estimates) between the corresponding person parameters of the BRM and the DDRM. A high correlation would indicate convergent validity for the two models and, consequently, for the two item formats.

Our third aim focuses on advantages of the DDRM over the use of raw mean scores. Specifically, we investigate whether correlational patterns of the two dimensions of core interest (i.e., location and expansion) differ when relying either on manifest mean scores or on latent parameter estimates. First, we again consider the correlation of the location estimates of the RS1 and the RS2 format, expecting higher convergent validity for the model parameters than for mean scores. Second, we assess whether the scale-inherent correlation among the two dimensions expansion and location is smaller for the model-based than the descriptive estimates. For this purpose, concerning the manifest mean scores, we focus on the correlation of the interval width and the absolute deviance of the response-interval location from the scale midpoint. Concerning the model parameters, this corresponds to the correlation of the person expansion parameter and the absolute value of the person location parameter. Higher convergent validity and a smaller internal correlation among the two dimensions would justify the employment of the proposed model.

In the following, we outline the BRM (Noel & Dauvier, 2007; Noel, 2014) in Sect. 2 and subsequently extend the model to the DDRM in Sect. 3. Next, we present a simulation study for the DDRM in Sect. 4. In Sect. 5 we report an empirical example in which we model both RS1 and RS2 responses using a joint hierarchical model that incorporates both the BRM and DDRM. We finally discuss the implications and limitations of the proposed model in Sect. 6.

## The Beta Response Model (BRM)

As a running example, we use the response scale implemented in our empirical example, which allows respondents to select values from 0 to 100. To fit the BRM, the observed responses $X^*$ must first be rescaled using the transformation $X= \tfrac{X^*+1}{102}$ so that $X \in (0,1)$. This is required for computational reasons as response values must not be equal to 0 or 1, thereby ensuring that the log-likelihood does not become $-\infty$ (see Stan Development Team, 2022).^1^

In a standard testing scenario, the random variable $X_{ij}$ represents the response of a respondent $i = 1,\dots , I$ (number of respondents) on item $j = 1, \dots , J$ (number of items). Noel and Dauvier (2007) derived $X_{ij}$ by proposing the following theoretical response mechanism: The respondent assigns a proximity judgment to each of the semantically anchored endpoints of the response scale, resulting in two psychological values, namely, $\upsilon _{ij}^{(A)}$ for agreement and $\upsilon _{ij}^{(D)}$ for disagreement. To generate a single response, both values are interpolated into a relative proportion on the response scale,

1 $$\begin{aligned} X_{ij}= \frac{\upsilon _{ij}^{(A)}}{\upsilon _{ij}^{(D)}+\upsilon _{ij}^{(A)}}. \end{aligned}$$

The resulting response variable $X_{ij}$ denotes the degree of agreement on the unit-scale segment. Both $\upsilon _{ij}^{(A)}$ and $\upsilon _{ij}^{(D)}$ are assumed to be positive values and are modeled as gamma-distributed random variables with separate shape parameters $m_{ij}$ and $n_{ij}$, but a common scale parameter *s*,

$$\begin{aligned} \upsilon _{ij}^{(A)}&\sim \Gamma (m_{ij},s), \\ \upsilon _{ij}^{(D)}&\sim \Gamma (n_{ij},s). \end{aligned}$$

This is an arbitrary yet advantageous choice since it implies that the response variable $X_{ij}$ follows a beta distribution (Johnson et al., 1995),

2 $$\begin{aligned} X_{ij} \sim \text {Beta}(m_{ij},n_{ij}). \end{aligned}$$

To transform the beta distribution into an IRT model, the shape parameters $m_{ij}$ and $n_{ij}$ are reparameterized in terms of a latent person ability $\theta _{i}$, a latent item difficulty $\delta _{j}$, an item precision parameter $\tau _{j} \ge 0$, and a general scaling parameter $\alpha > 0$. A slightly modified version of the original parameterization^2^ is given by,

3 $$\begin{aligned} m_{ij}&= \exp [ \alpha (\theta _{i} - \delta _{j}) + \tau _{j}], \nonumber \\ n_{ij}&= \exp [-\alpha (\theta _{i} - \delta _{j}) + \tau _{j}]. \end{aligned}$$

The positive versus negative sign for $\pm \alpha$ has the effect that differences between ability and difficulty parameters (i.e., $\theta _{i} - \delta _{j}$) result in parameters $m_{ij}$ and $n_{ij}$ of the beta distribution that are further away from the value 1 in opposite directions (while assuming $\tau _j = 0$). Depending on the sign of the difference $\theta _{i} - \delta _{j}$, the mode of the beta distribution moves up or down on the response scale, thereby resulting in answers that indicate agreement or disagreement on the response scale, respectively. Since the variance of the beta distribution decreases when both parameters $m_{ij}$ and $n_{ij}$ increase,^3^ larger values of $\tau _{j}$ result in a steeper response-density curve, and thus, in less variability of the observed responses.

## The Dirichlet Dual Response Model (DDRM)

### Model Structure

The BRM is concerned with a continuous bounded scale and is based on the idea that each response divides the scale into two proportions that sum up to one. Analogously, the RS2 can be viewed as a continuous bounded scale where each response interval divides the scale into *three* proportions. A Dirichlet distribution with three parameters can thus be applied to the RS2 format, similar to the beta distribution with two parameters for the RS1 format. In fact, Noel (2014) already used a Dirichlet distribution to derive an extended version of the BRM, the beta unfolding model that applies to single continuous responses. Building on this approach, we develop a different parameterization that applies to the RS2 format.

A response interval can be described by two values, namely, $Y^*_{L}$ for the lower bound (adjusted via the left slider), and $Y^*_{U}$ for the upper bound (adjusted via the right slider). Due to the same computational reasons as for the BRM, the original responses on the scale from 0 to 100 are first transformed to avoid values at the boundaries of the response scale (see Stan Development Team, 2022). Since respondents can select identical values for both sliders in the RS2 format (resulting in an response interval of length zero), it is also necessary to ensure that $Y_{L}$ is strictly smaller than $Y_{U}$. As a remedy, the transformations $Y_{L} = \tfrac{Y^*_{L} + 1}{103}$ and $Y_{U} = \tfrac{Y^*_{U} + 2}{103}$ ensure that the strict inequalities $0< Y_{L}< Y_{U} < 1$ hold.

Using the transformed responses, we define a response vector $\varvec{Y}$ which contains the three proportions describing the response interval on a unit scale,

4 $$\begin{aligned} \varvec{Y} = \begin{pmatrix} Y_{L} \\ Y_{U} - Y_{L}\\ 1 - Y_{U} \end{pmatrix}. \end{aligned}$$

In this vector, ${Y}_L$ is the proportion to the left of the response interval, ${Y}_{U}-Y_L$ is the middle proportion (i.e., the relative width of the response interval), and $1-{Y}_{U}$ is the proportion to the right of the response interval.

For the DDRM, we extend the response mechanism assumed by the BRM (Noel & Dauvier, 2007). The response vector $\varvec{Y}_{ij}$ for respondent *i* answering item *j* is modeled by an interpolation mechanism of the three latent values $\upsilon _{ij}^{(A)}$, $\upsilon _{ij}^{(E)}$, and $\upsilon _{ij}^{(D)}$,

5 $$\begin{aligned} \varvec{Y}_{ij} = \Bigg ( \frac{\upsilon _{ij}^{(A)}}{\upsilon _{ij}^{(A)} + \upsilon _{ij}^{(E)} + \upsilon _{ij}^{(D)}}, \frac{\upsilon _{ij}^{(E)}}{\upsilon _{ij}^{(A)} + \upsilon _{ij}^{(E)} + \upsilon _{ij}^{(D)}}, \frac{\upsilon _{ij}^{(D)}}{\upsilon _{ij}^{(A)} + \upsilon _{ij}^{(E)} + \upsilon _{ij}^{(D)}} \Bigg )'. \end{aligned}$$

The latent value $\upsilon _{ij}^{(A)}$ reflects overall agreement with an item since larger values lead to an increase of the leftmost proportion and to a decrease of the other two proportions, which in turn shifts the response interval to the right side of the scale (i.e., in the direction of agreement). The latent value $\upsilon _{ij}^{(D)}$ reflects overall disagreement and follows a similar mechanism, but in the opposite direction. Finally, the parameter $\upsilon _{ij}^{(E)}$ represents the expansion of latent values, that is, the variability of latent agreement and disagreement values. If $\upsilon _{ij}^{(E)}$ increases, the middle proportion becomes larger whereas the two outer proportions become smaller, in turn leading to a wider response interval.

Similar to the BRM, the three latent values are assumed to be gamma-distributed with a common scale parameter *s* (Noel, 2014). Concerning the shape parameters, $a_{ij}$ and $d_{ij}$ again reflect agreement and disagreement, respectively, whereas $e_{ij}$ refers to the expansion of latent values,

6 $$\begin{aligned} \upsilon _{ij}^{(A)}&\sim \Gamma (a_{ij},s), \nonumber \\ \upsilon _{ij}^{(E)}&\sim \Gamma (e_{ij},s), \nonumber \\ \upsilon _{ij}^{(D)}&\sim \Gamma (d_{ij},s). \end{aligned}$$

Equations (5) and (6) imply that the response vector follows a Dirichlet distribution,

7 $$\begin{aligned} \varvec{Y}_{ij} \sim \text {Dir}(a_{ij},e_{ij},d_{ij} ), \end{aligned}$$

where the density function of the Dirichlet distribution is given by

8 $$\begin{aligned} f(\varvec{y}_{ij} | a_{ij}, e_{ij}, d_{ij}) = \frac{\Gamma (a_{ij} + e_{ij} + d_{ij}) }{ \Gamma (a_{ij}) \cdot \Gamma (e_{ij}) \cdot \Gamma (d_{ij}) } y_{ij1}^{a_{ij} - 1} y_{ij2}^{e_{ij} - 1} y_{ij3}^{d_{ij} - 1}. \end{aligned}$$

The Dirichlet distribution of the response vector ${\varvec{Y}}_{ij}$ is re-parameterized in terms of person and item parameters, thus building an IRT structure on top of the Dirichlet parameters,

9 $$\begin{aligned} a_{ij}&= \exp \bigl [ \alpha _{\lambda } (\theta _{i} - \delta _{j}) + \tau _{j}\bigr ], \nonumber \\ e_{ij}&= \exp \bigl [ \alpha _{\epsilon }(\eta _{i} + \gamma _{j}) + \tau _{j}\bigr ], \nonumber \\ d_{ij}&= \exp \bigl [-\alpha _{\lambda }(\theta _{i} - \delta _{j}) + \tau _{j}\bigr ]. \end{aligned}$$

Note that some of the parameters appear in both the BRM and the DDRM (e.g., $\theta _{i}$ or $\delta _{j}$). Formally, these parameters fulfill different roles depending on the specific model structure. Substantively, however, these parameters have an equivalent interpretation in the BRM and the DDRM, and thus, we use the same letters to facilitate readability. In the empirical example, where both models are analyzed jointly, we label these corresponding parameters using upper scripts *B* for the BRM (e.g., $\theta ^{B}_{i}$) and *D* for the DDRM (e.g., $\theta ^{D}_{i}$).

In the DDRM, the latent parameterization of agreement $a_{ij}$ and disagreement $d_{ij}$ follows a similar mechanism as for $m_{ij}$ and $n_{ij}$, respectively, in the BRM. Essentially, the difference in person and item parameters (i.e., $\theta _{i} - \delta _{j}$) moves the response interval up or down on the response scale, thus reflecting the central tendency of the distribution of latent values. The latent expansion value $e_{ij}$ controls the width of the response interval and is parameterized in terms of a person parameter $\eta _{i}$ and an item parameter $\gamma _{j}$. The parameter $\eta _i$ refers to a respondent’s tendency to provide wide response intervals, which may represent various psychological constructs such as variability in the latent trait or behavior, subjective uncertainty, or response styles. The expansion parameter $\gamma _j$ represents an item’s tendency to elicit wide versus narrow response intervals. Parameters $\eta _{i}$ and $\gamma _j$ are combined by summation to obtain $e_{ij}$, which contrasts with the subtraction used for the latent location dimension (i.e., $\theta _{i} - \delta _{j}$ for $a_{ij}$ and $d_{ij}$). Using the sum of the person and item parameters (i.e., $\eta _{i} + \gamma _{j}$) results in a more intuitive interpretation, as for both parameters larger values then correspond to wider response intervals.

The parameter $\tau _{j}$ fulfills an equivalent function as in the BRM, representing the precision of responses both on the location and the expansion dimension at the same time. Essentially, large values of $\tau _{j}$ imply that respondents provide consistent response intervals in terms of locations and widths. Lastly, we assume a separate scaling parameter for each latent dimension, that is, $\pm \alpha _{\lambda }$ for the location dimension and $\alpha _{\epsilon }$ for the expansion dimension. In the location dimension, the parameter $\alpha _\lambda$ serves the same function as in the BRM: it allows for a scaling of the difference between person ability and item difficulty (i.e., $\theta _{i} - \delta _{j}$), and thereby facilitates shifts of the whole response interval up and down on the response scale. In the expansion dimension, the scaling parameter $\alpha _\epsilon$ only controls the influence of the corresponding person and item parameters (i.e., $\eta _{i} + \gamma _{j}$).

**Fig. 2:**
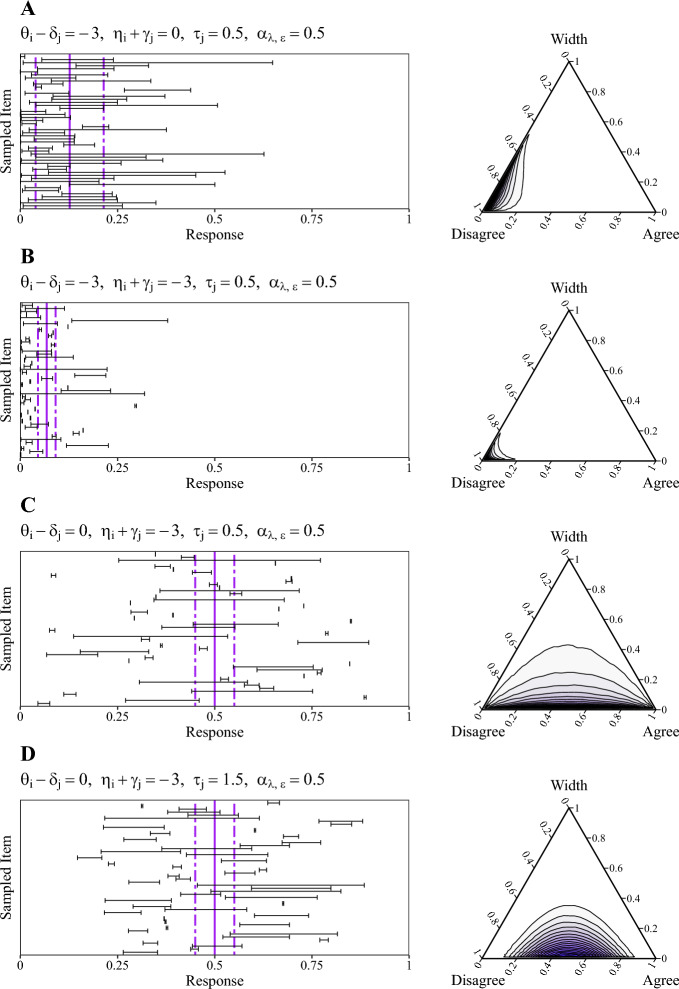
Response distributions and sampled interval responses for the DDRM. *Note*. The left column shows 50 randomly drawn response intervals that correspond to the Dirichlet distributions illustrated in the right column (with densities approximated based on 100,000 random draws). Solid vertical lines show the expected value for the midpoint $(Y_L + Y_U) / 2$ of the response interval (i.e. expected location), whereas the dashed vertical lines show the expected values for the corresponding lower bound and upper bound (i.e., $Y_L$ and $Y_U$, respectively).

Figure 2 shows four exemplary Dirichlet distributions of interval responses using ternary plots (right column) for different configurations of the latent parameters, including 50 randomly drawn response intervals for each scenario (left column). As intended, the location and expansion parameters clearly affect the expected interval location (solid vertical line) and expected interval width (dashed vertical lines), respectively. However, locations and widths are not exclusively influenced by the corresponding latent dimension, but are also affected by the respective other dimension. When comparing Fig. 2A and B, we see that a change in $\eta _i - \gamma _j$ (i.e., the expansion dimension) causes a shift in the expected interval location. Analogously, when comparing Fig. 2B and C, we see that a change in $\theta _i - \delta _{j}$ (i.e., the location dimension) causes a shift in the expected interval width. This behavior is due to the fact that the DDRM accounts for the inherent dependency of interval location and width on the bounded response scale. Also, note that a change in $\tau _j$ (precision) does not cause a change in the expected interval width. Instead, larger values of $\tau _j$ imply that response intervals are more homogeneous both with respect to their locations and widths (see Fig. 2C, D).

### Item Information

**Fig. 3:**
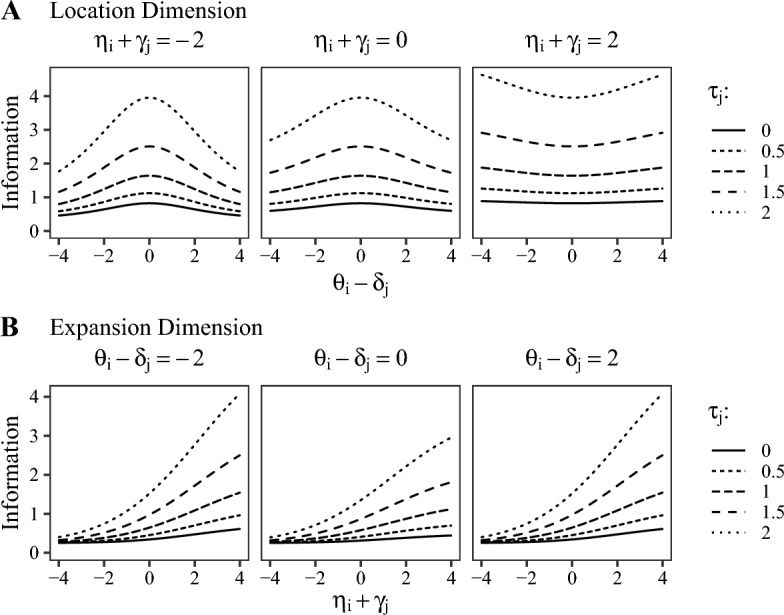
Item information for the person parameters of the DDRM. *Note*. Scaling parameters $\alpha _{\lambda }$ and $\alpha _{\epsilon }$ are fixed to 0.5.

To investigate the model’s sensitivity to changes in the latent parameters, we derived the item-information functions for $\theta _i$ and $\eta _i$ based on the expected Fisher information. For a full derivation of the log-likelihood, first and second derivatives, and item information, see Appendix B. The item information for $\theta _i$ is illustrated in Fig. 3A and given by

10 $$\begin{aligned} \mathcal I_{\theta }&= -\mathbb E \Biggl [ \frac{\partial ^2 \ln L(\Theta ; \varvec{Y})}{\partial ^2 \theta _{i}} \Biggl ] \nonumber \\ {}&= -\Bigl [ (\zeta _a^{(a)} \alpha _{\lambda } a_{ij}) + (- \zeta _d^{(a)} \alpha _{\lambda } d_{ij}) \Bigr ] \alpha _{\lambda } a_{ij} \nonumber \\ {}&\quad -\Bigl [ (\zeta _a^{(d)} \alpha _{\lambda } a_{ij}) + (- \zeta _d^{(d)} \alpha _{\lambda } d_{ij}) \Bigr ] (- \alpha _{\lambda }) d_{ij} \end{aligned}$$

with

11 $$\begin{aligned} \zeta _a&= \Bigl [ \psi (a_{ij} + e_{ij} + d_{ij}) - \psi (a_{ij}) + \ln (y_{ij1}) \Bigr ], \nonumber \\ \zeta _d&= \Bigl [ \psi (a_{ij} + e_{ij} + d_{ij}) - \psi (d_{ij}) + \ln (y_{ij3}) \Bigr ], \nonumber \\ \zeta _a^{(a)}&= \frac{\partial \zeta _a}{\partial a_{ij}} = \psi '(a_{ij} + e_{ij} + d_{ij}) - \psi '(a_{ij}), \nonumber \\ \zeta _d^{(d)}&= \frac{\partial \zeta _d}{\partial d_{ij}} = \psi '(a_{ij} + e_{ij} + d_{ij}) - \psi '(d_{ij}), \nonumber \\ \zeta _a^{(d)}&= \frac{\partial \zeta _a}{\partial d_{ij}} = \psi '(a_{ij} + e_{ij} + d_{ij}),\nonumber \\ \zeta _d^{(a)}&= \frac{\partial \zeta _d}{\partial a_{ij}} = \psi '(a_{ij} + e_{ij} + d_{ij}), \end{aligned}$$

where $\psi '(x) = \partial ^2 \ln \Gamma (x) / \partial ^2 x$ is the trigamma function. The item information for $\eta _i$ is illustrated in Fig. 3B and is given by

12 $$\begin{aligned} \mathcal I_{\eta }&= -\mathbb E \Biggl [ \frac{\partial ^2 \ln L(\Theta ; \varvec{Y})}{\partial ^2 \eta _{i}} \Biggl ] \nonumber \\ {}&= - \Bigl [\psi '(a_{ij} + e_{ij} + d_{ij}) - \psi '(e_{ij})\Bigr ] \alpha _{\epsilon }^{2} e^2_{ij}. \end{aligned}$$

The item-information curves for the location parameter $\theta _i$ (Fig. 3A) under the condition of small values for the expansion dimension ($\eta _i + \gamma _j$; Fig. 3A, left panel) are unimodal. The shape of these functions is very similar to the item-information curves derived for the BRM by Noel and Dauvier (2007). With higher values of $\eta _i + \gamma _j$ (see Fig. 3A, middle and right panel), the curves tend towards bimodal U-shapes. For an arbitrary $\tau _j$ (i.e., a specific line type in the figure) the overall item-information increases when $\eta _i + \gamma _j$ increases (compare all panels of Fig. 3A from left to right), except for the point $\theta _i - \delta _j = 0$; the item-information at that point stays constant for increasing $\eta _i + \gamma _j$. This behavior is caused by the asymmetric model architecture: $\eta _i + \gamma _j$ raises or lowers the sum of the Dirichlet parameters (i.e., $a_{ij}, e_{ij}, d_{ij}$) independently from $\theta _i - \delta _j$. Thus it can govern the precision of the corresponding response distribution without a change in $\theta _i - \delta _j$.

In line with this mechanism, the item-information curves for the expansion parameter $\eta _i$ (Fig. 3B) are monotonically increasing for all three levels of the location dimension (i.e., $\theta _i-\delta _j$). For lower levels of $\eta _i + \gamma _j$, item information is generally lower, while the overall information level is raised by moving the location dimension away from zero (i.e., $|\theta _i - \delta _j| > 0$; comparing the middle panel of Fig. 3B to the outer ones). Again, the reason is that $\theta _i - \delta _j$ raises or lowers the sum of the Dirichlet parameters (i.e., $a_{ij}, e_{ij}, d_{ij}$) independently from $\eta _i + \gamma _j$. Since the sign of the scaling parameter $\alpha _{\lambda }$ differs for $a_{ij}$ and $d_{ij}$, $\theta _i - \delta _j = 0$ leads to the minimum precision of the distribution and consequently also to the lowest overall level of item information (see middle panel of Fig. 3B). The monotonically increasing item-information curve implies that the model is relatively insensitive to changes of latent parameters in the lower range of the expansion dimension. At the same time, the model is more sensitive when the location dimension is situated in the higher or lower region (i.e., away from zero). The item-information curve thus implies that response intervals are more informative when the interval width is large, and also, when the interval is located closer to one of the ends of the response scale.

## Simulation Study

### Data Generation

To investigate the parameter recovery of the DDRM, we conducted a simulation study. All R scripts are available at the Open Science Framework (https://osf.io/br8fa/). We simulated 300 datasets for $4\times 3$ conditions, namely, four different numbers of items ($J = 10, 15, 20, 30$) crossed with three different sample sizes ($I = 100, 250, 500$). The data-generating person parameters $\theta _i$ and $\eta _i$ were drawn from $\mathcal {N}(0,1)$ for each simulated dataset. In contrast, $\delta _j$ and $\gamma _j$ were randomly drawn from a fixed set of values given by the sequence from $[-2,2]$ with step size $\tfrac{4}{J}$. Thereby, we randomized the combinations of both parameters for each item across simulated datasets and items. Precision parameters $\tau _j$ were drawn from a uniform distribution, $\mathcal {U}(0,2)$, whereas scaling parameters $\alpha _{\lambda ,\epsilon }$ were fixed to 0.5 for all simulated datasets.

### Bayesian Parameter Estimation

The model was fitted to all simulated datasets in a Bayesian framework using Stan (Stan Development Team, 2021). To ensure identifiability, we implemented the model with a standard normal prior on the person parameters, thus fixing the group-level means to zero and the standard deviations to one,

13 $$\begin{aligned} \theta _i, \eta _i \sim \mathcal {N}(0,1). \end{aligned}$$

To limit computation times and avoid divergent transitions of the sampler, we chose weakly informative priors for the remaining parameters,^4^

14 $$\begin{aligned} \delta _j&\sim \mathcal {N}(\mu _{\delta },\sigma _{\delta }), \nonumber \\ \gamma _j&\sim \mathcal {N}(\mu _{\gamma },\sigma _{\gamma }), \nonumber \\ \mu _{\delta }, \mu _{\gamma }&\sim \mathcal {N}(0,1.5), \nonumber \\ \sigma _{\delta }, \sigma _{\gamma }&\sim \Gamma (1.5,1.5), \nonumber \\ \tau _j&\sim \mathcal {N}(\mu _{\tau },\sigma _{\tau }) \text { truncated to } (0,\infty ), \nonumber \\ \mu _{\tau },\sigma _{\tau }&\sim \Gamma (1.5,1.5), \nonumber \\ \alpha _{\lambda }, \alpha _{\epsilon }&\sim \Gamma (1.5,1.5). \end{aligned}$$

We fitted the DDRM in *R* (R Core Team, 2021) with *Stan* (Stan Development Team, 2021) via the *CmdStanR* package (Gabry & Češnovar, 2021) by running four chains of the Hamiltonian-Monte-Carlo (HMC; Betancourt, 2018) no-U-turn sampler (NUTS). Each chain included 500 burn-in iterations and 3,500 sampling iterations, resulting in a total of 14,000 samples per parameter. Concerning convergence of the sampler, there were overall 17 model fits across five conditions that had divergent transitions of the HMC chains. We excluded these model fits from further analyses. We further excluded one model fit for high values of the $\widehat{R}$ statistic ($> 1.05$; Vehtari et al., 2021). For the remaining model fits, all parameters had an $\widehat{R} < 1.03$. Concerning the effective sample sizes (ESS), the bulk ESS, which determines the precision of the estimated posterior means or medians, as well as the tail-ESS, which determines the precision of the estimated lower and upper credibility bounds, were satisfactory for all models and parameters (minimum bulk-ESS across model fits: minimum = 212, median = 1,002; minimum tail-ESS across model fits: minimum = 428; median = 2,693).

### Performance Measures

We used the posterior medians as point estimates for the parameters. Based on these estimates, we computed several measures of parameter-recovery performance for each group of parameters (e.g., using the $\theta _i$ parameters of all individuals), which were then averaged over the 300 model fits within each condition. As performance measures, we focus on the correlations between estimated and true parameters (referred to as correlation), the mean signed difference (bias), the root mean square error (RMSE), and the percentage of $95\%$ highest density intervals (HDIs) covering the true parameter value (coverage).

### Results and Discussion

**Fig. 4:**
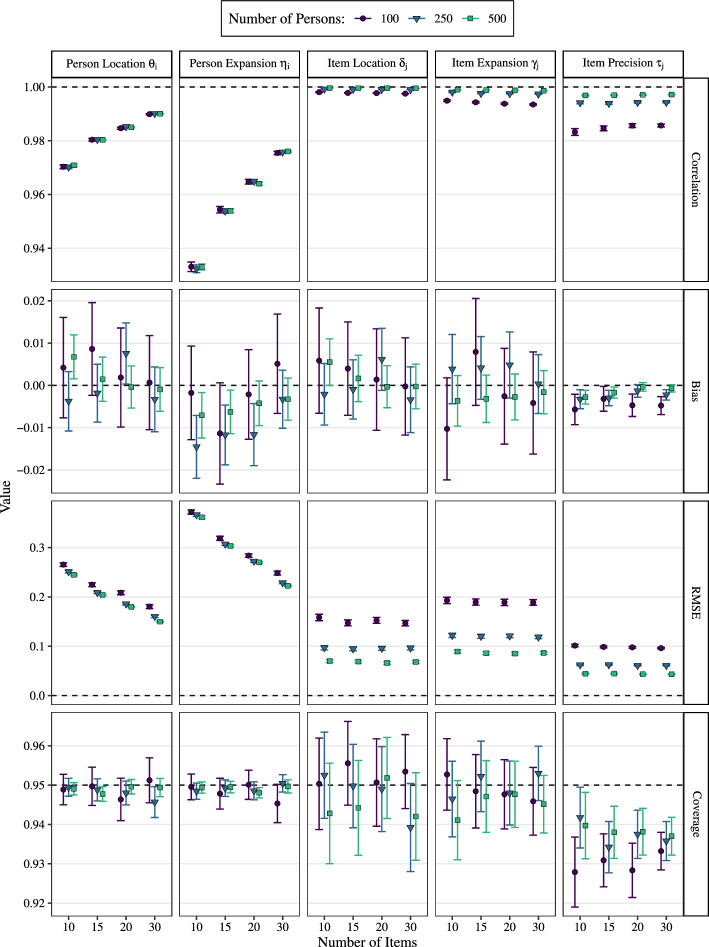
Average performance measures for the DDRM parameters. *Note*. Performance measures were computed for each group of parameters separately (i.e., $\theta _i$, $\eta _i$, etc.) and then averaged across the 300 replications. Error bars show corresponding $95\%$ confidence intervals.

Figure 4 shows the different performance measures (rows) for each group of parameters (columns). The bias estimates (second row) are overall negligibly small and, with the exception of $\eta _i$ and $\tau _j$, which were overall slightly underestimated, basically reduce to noise. The estimates for correlation (first row) and RMSE (third row) reveal that higher numbers of items benefit the person-parameter estimates while higher numbers of persons benefit the item-parameter estimates. Additionally, we see that the parameters concerning the location dimension ($\theta _i$, $\delta _j$) show a lower RMSE than the corresponding parameters concerning the expansion dimension ($\eta _i$, $\gamma _j$). This trend is especially pronounced for person parameters. To achieve a performance of the person expansion $\eta _i$ comparable to the performance of the person location $\theta _i$ using 10 items, it would be necessary to double the number of items. Given the lower item information for $\eta _i$ (see Fig. 3), this is not surprising but should be considered when deciding on a certain test length. Although larger numbers of persons and items obviously lead to higher precision in parameter estimates, there are diminishing returns on investment when stepping up from 250 to 500 persons or from 20 to 30 items. Comparing the item parameters, the recovery of precision parameters $\tau _j$ was considerably worse than for the other two parameters. Besides the mentioned negative bias and lower correlation, $\tau _j$ was the only parameter group that did not achieve the targeted coverage across all conditions, which is a consequence of the negative bias.

We also used the simulated datasets to assess the added value of the rather complex DDRM by comparing the performance of raw mean scores and latent person parameters. Specifically, we focused on a critical property of continuous bounded interval responses, namely, the scale-inherent dependence of interval locations and interval widths. The further away a response interval is placed from the scale midpoint, the smaller the maximum possible width becomes, which in turn implies a negative correlation. We assessed this dependence by computing the absolute deviance from the scale midpoint (ADSM) as an alternative representation of a given response interval,

15 $$\begin{aligned} Y_{\text {ADSM}} = |0.5 - \tfrac{Y_{L} + Y_{U}}{2} |. \end{aligned}$$

For each simulated dataset, we then computed the correlation between the individual mean scores for the response-interval width and the mean scores for the absolute deviance from the scale midpoint, $Y_{\text {ADSM}}$. Averaged across all 12 conditions and all replications, this correlation was $r = -.74$ ($95\%$ CI = $[-.82,-.66]$), indicating a strong dependence of location and width. Analogously to the manifest responses, we computed the correlation for the latent parameters of the DDRM while focusing on their absolute values for the location dimension (i.e., $|\theta _i|$ and $\eta _j$). Contrary to the raw mean scores, the mean correlation of recovered parameters was close to zero, $r = -.01$ ($95\%$ CI = $[-.16,.13]$). These values are very close to the mean correlation between the true generating parameters ($r =.00$, $95\%$ CI = $[-.14,.14]$). Overall, these results show that the raw mean scores for interval location and width exhibit a strong negative correlation even when the true, data-generating parameters are basically uncorrelated. This is a major drawback of using simple mean scores for response intervals. As a remedy, the DDRM provides parameter estimates for location and expansion with a correlation close to zero, which facilitates the estimation of the actual, data-generating parameter structure. We will come back to this point in the context of the empirical example.

## Empirical Example

### Sample and Procedure

The primary goal of our empirical study was the collection of a suitable data set for the development and evaluation of the DDRM. The secondary goal was to compare the DDRM location parameters to those of the BRM. In an effort to maximize the number of items and respondents, we decided to split neither the sample nor the item pool. Instead, for the standard single-range-slider format (RS1), we used a different set of items from an established measurement instrument (Danner et al., 2019). While this approach does not allow us to perform a direct comparison of the two response formats at the item level, we can still compare the person location parameters of the DDRM and the BRM since both parameters reflect the central tendency of the same trait. Moreover, a test of the convergent validity at the person level with distinct items per response format provides an even stricter test than the alternative approach of using an identical set of items with repeated measurement.

We conducted an online survey containing 36 RS2 items and 12 RS1 items.^5^ Recovery simulations based on a previous version of the DDRM showed that sufficiently precise parameter estimates could be obtained with a sample size of $N = 200$. The original sample consisted of 246 German-speaking respondents of which the majority were psychology students. In total, 24 respondents were excluded as they provided extremely long response times ($n = 3$), univariate extreme responses ($n = 6$), or multivariate extreme responses ($n = 15$). The final sample consisted of 222 respondents (female: 140, male: 80, diverse: 2) with a median age of 27 years ($M = 29.4, \textit{SD} = 10.9$).

The items were presented in two blocks. First, 36 Extraversion items from the International Personality Item Pool (IPIP; Goldberg, 1999) had to be answered using the RS2 format. Second, 12 Extraversion items from the Big Five Inventory 2 (BFI-2; Danner et al., 2019) had to be answered using the RS1 format. Regarding the RS2 items, respondents were instructed to indicate how well the presented statement applied to themselves (e.g., “I like to visit new places”). In doing so, they had to use the two sliders to specify a range of values indicating the variability of the statement’s fit across different situations (including both work and private life). Whereas broader response intervals had to be specified for statements with a high variability of fit across situations, narrower response intervals had to be chosen if the fit of the statement was similar across different situations. Respondents were also instructed to consider only typical behaviors while disregarding extreme situations. In the instructions for the RS1 items, respondents were merely asked to indicate how well the statement applied to themselves by choosing a single value on the response scale. Both the RS1 and the RS2 format were verbally and numerically anchored at their endpoints ($0 = \textit{does not apply at all}$, $100 = \textit{fully applies}$), while the midpoint (50) was also labeled on the scale (see Fig. 1). Above each of the adjustable visual sliders, the currently specified numeric value was displayed. The initial values for the sliders were 50 for the RS1 and [0, 100] for the RS2. The sliders had to be moved at least once before respondents could proceed to the next item. Items were presented one at a time and in random order within each block.

### Measures

#### IPIP-NEO

The scale contained 36 Items from the IPIP-NEO (Goldberg, 1999) in our own German translation. We selected items representing the core of the Extraversion factor in a multidimensional graded response model (Samejima, 1969; Chalmers, 2012).^6^ The selected items mainly belonged to the facets Sociability, Activity Level, Adventurousness, Positive Emotions, and Unrestraint. McDonald’s $\omega _t$ (internal consistency) was .94 in our sample for the response-interval locations, and .92 in the original Eugene Springfield Community Sample (ESCS; Goldberg, 1999), which used a 5-point Likert-type scale and included 570 respondents (female: 330, male: 240) with ages ranging from 20 to 85 years. McDonald’s $\omega _h$ (g-saturation) was .63 for our sample and .62 for the ESCS. Hence, our subset of IPIP-NEO items which were answered in the RS2 format performed equally well in our study as in the original study, despite differences in item selection, item format, and translation.

#### BFI-2

The 12 items of the Extraversion scale from the German version of the BFI-2 (Danner et al., 2019; Soto & John, 2017) cover three facets: Sociability, Assertiveness, and Energy Level. In our sample, McDonald’s $\omega _t$ and $\omega _h$ for the RS1 format were .92 and .79, respectively. The latter value resembles McDonald’s $\omega _h =.80$ obtained with 5-point Likert-type items in the original norming sample which consisted of 770 respondents (female: 396, male: 374) with a mean age of 44.5 years ($SD = 13.8$). This shows that the BFI-2 performed equally well in our study as in the original study, which provides evidence for the measurement quality of the RS1 format.

### Bayesian Parameter Estimation

To address research questions regarding the correlation of person parameters across different response formats, it is convenient to combine the BRM and the DDRM into a joint model. For this purpose, we assumed a multivariate normal prior distribution for the person parameters of both models (upperscripts *B* and *D* stand for the BRM and DDRM, respectively),

16 $$\begin{aligned} (\theta ^{B}_i, \theta ^{D}_i, \eta ^D_i) \sim \mathcal {MVN} (\varvec{\mu }, \varvec{\Sigma }). \end{aligned}$$

The covariance matrix $\varvec{\Sigma }$ was parameterized in terms of a correlation matrix and a vector of standard deviations,

17 $$\begin{aligned} \varvec{\Sigma }= \text {diag} (\varvec{\sigma }) \,\varvec{\Omega }\, \text {diag} (\varvec{\sigma }). \end{aligned}$$

The Cholesky factor decomposition of the correlation matrix (Barnard et al., 2000) was used to assume an uninformative LKJ-Cholesky prior (Lewandowski et al., 2009),

18 $$\begin{aligned} \varvec{\Omega }&= \varvec{\Omega }_L \varvec{\Omega }_L^T, \nonumber \\ \varvec{\Omega }_L&\sim \text {LKJ-Cholesky}(1). \end{aligned}$$

To ensure the identifiability of the hierarchical model, we fixed the group-level means to $\varvec{\mu }= {\varvec{0}}$ and the standard deviations to $\varvec{\sigma }= {\varvec{1}}$.

For the item parameters, we assigned normal priors to $\delta _j$ and $\gamma _j$, and truncated normal priors to $\tau _j$ along with weakly informative hyperpriors. For all $\alpha$ parameters we specified a weakly informative truncated Student-t prior. Since the priors apply to both the BRM and the DDRM, we drop the superscripts,^7^

19 $$\begin{aligned} \delta _j&\sim \mathcal {N}(\mu _{\delta },\sigma _{\delta }), \nonumber \\ \gamma _j&\sim \mathcal {N}(\mu _{\gamma },\sigma _{\gamma }), \nonumber \\ \mu _{\delta }, \mu _{\gamma }&\sim \text {t}(3,0,2), \nonumber \\ \sigma _{\delta }, \sigma _{\gamma }&\sim \text {t}(3,0,2) \text { truncated to }(0,\infty ), \nonumber \\ \tau _j&\sim \mathcal {N}(\mu _{\tau },\sigma _{\tau })\text { truncated to }(0,\infty ), \nonumber \\ \mu _{\tau }&\sim \text {t}(3,0,2) \text { truncated to }(0,\infty ), \nonumber \\ \sigma _{\tau }&\sim \text {t}(3,0,2) \text { truncated to }(0,\infty ), \nonumber \\ \alpha , \alpha _{\lambda }, \alpha _{\epsilon }&\sim \text {t}(3,0,2) \text { truncated to }(0,\infty ). \end{aligned}$$

We fitted the Bayesian hierarchical model using the same software as for the simulation study (see Sect. 4).^8^ We ran 4 chains of Stan’s HMC NUTS sampler, each with 4,000 burn-in and 4,000 sampling iterations, and a thinning factor of 2, resulting in 8,000 samples per parameter.^9^ We checked convergence of the chains via the diagnostic function of the CmdStanR package (Gabry & Češnovar, 2021) and via the convergence statistics split $\widehat{R}$ and effective sample size (ESS; Vehtari et al., 2021). All $\widehat{R}$ were smaller than 1.01, the minimum bulk-ESS was 2, 828 and the minimum tail-ESS was 4, 358, which indicated convergence of all HMC chains. Also, there were no divergent transitions for any of the chains.

### Results and Discussion

#### Descriptive Statistics

There were no missing data. If respondents answered an item multiple times by going back to previous pages of the survey, only the first response was used for analysis. The means of all RS1 responses ($M = 58.67, \textit{SD} = 24.99$) and all interval locations in the RS2 format ($M = 56.65, \textit{SD} = 24.96$) were comparable. The mean interval width was about $25\%$ of the scale segment’s length ($M = 26.12$, $\textit{SD} = 15.97$). Regarding mean scores, the RS2 interval locations had a more balanced variance ratio of person statistics to item statistics ($\tfrac{SD_{person}}{SD_{item}} = \tfrac{12.27}{9.35} = 1.31$) compared to the RS1 ($\tfrac{SD_{person}}{SD_{item}} = \tfrac{16.45}{5.59} = 2.94$), which could be beneficial for parameter estimation. However, the fact that the variance ratio was closer to one for RS2 than RS1 might also be due to the larger number of items for the RS2 format. The variance ratio was even more unbalanced for the RS2 interval widths ($\tfrac{SD_{person}}{SD_{item}} = \tfrac{9.84}{2.34} = 4.2$), suggesting that items might not have differentiated very well in terms of interval widths.

Given that we transformed all raw responses by adding a certain smoothing constant to avoid proportion values of 0 and 1, it is of interest how many of the untransformed responses actually were at a boundary (meaning that one of the sliders hit the limits of the response scale or the other slider). At the level of respondents, for RS1 responses, the mean percentage of responses $X^* = 0$ was $1.43\%$ ($Q_{[.025,.975]} = [0,16.67]$) and the mean percentage of responses $X^* = 100$ was $5.83\%$ ($Q_{[.025,.975]} = [0,41.67]$). For RS2 responses, the mean percentage of responses with only $Y^*_L = 0$ was $2.63\%$ ($Q_{[.025,.975]} = [0,17.99]$) and the mean percentage of responses with only $Y^*_U = 100$ was $7.33\%$ ($Q_{[.025,.975]} = [0,41.67]$). On the other hand, the mean percentage of responses where only the interval width $Y^*_U - Y^*_L = 0$ was $1.58\%$ ($Q_{[.025,.975]} = [0,11.11]$) and the mean percentage of responses where the interval width $Y^*_U - Y^*_L = 100$ was $0.04\%$ ($Q_{[.025,.975]} = [0,0]$). Further, the mean percentage of responses where $Y^*_L = 0$ and $Y^*_U - Y^*_L = 0$ was $0.49\%$ ($Q_{[.025,.975]} = [0,5.56]$) and the mean percentage of responses where $Y^*_U = 100$ and $Y^*_U - Y^*_L = 0$ was $0.94\%$ ($Q_{[.025,.975]} = [0,9.65]$). Overall, the percentage of RS1 and RS2 responses at the boundaries was thus relatively low.

**Fig. 5:**
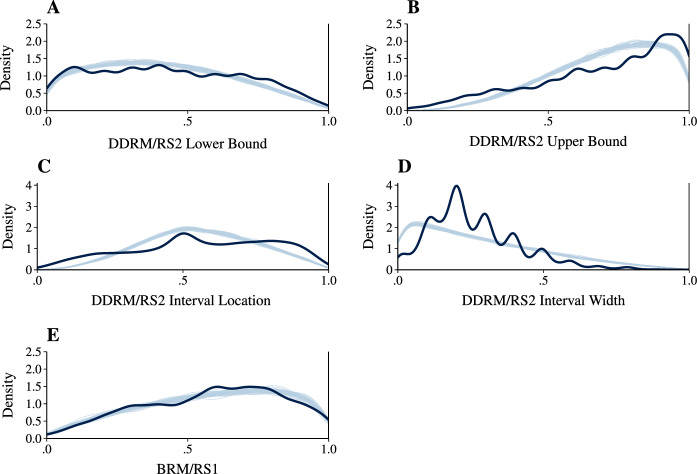
Marginal posterior predictive checks for the DDRM (Panels A–D) and the BRM (Panel E) *Note.* Dark-blue lines show the empirical distributions of responses. Light-blue lines correspond to posterior-predicted densities drawn from the DDRM or the BRM (50 densities per plot) (Color figure online).

#### Model Fit

The fit of Bayesian models can be evaluated via graphical checks (Gelman, Carlin, et al., 2014, Chapter 6; Gabry et al., 2019) by comparing the actual, empirical responses to posterior-predicted responses drawn from the fitted model. To facilitate an in-depth assessment of model fit, Fig. 5 shows a direct comparison of the empirical versus posterior-predicted densities with respect to five aspects of the data: the RS2 lower and upper bounds of the response interval, the RS2 interval locations and widths, as well as the RS1 responses. For the BRM (Fig. 5E), and for the lower bounds (Fig. 5A) and upper bounds (Fig. 5B) of the DDRM, posterior-predicted distributions fit the empirical data reasonably well. Regarding the upper bounds of the RS2, Fig. 5B shows that the empirical distribution is slightly shifted towards the upper end of the response scale compared to the distribution implied by the DDRM. In contrast, Fig. 5C shows that the DDRM predicts distributions of interval locations that are concentrated too much in the middle of the response scale. According to Fig. 5D, the model also predicts too narrow intervals (i.e., overly small widths). Consequently, the skew of the empirical and posterior-predicted distributions does not match. The plots also show that the respondents’ preferences for round figures (i.e., the distribution modes on the numbers 10, 20, etc.) were not accounted for by the models.

**Fig. 6:**
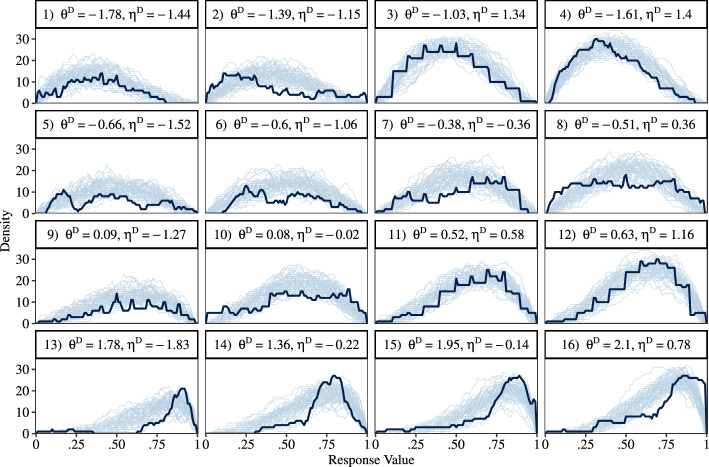
Posterior predictive checks: aggregated interval responses on the respondent level. *Note.* Dark-blue lines show the empirical distributions of aggregated interval responses. Light-blue lines correspond to posterior-predicted densities drawn from the DDRM (50 densities per plot). Panels are ordered by the magnitude of estimated parameter values for the corresponding respondent. First row: $\theta ^D < -1$. Second row: $-1 \le \theta ^D < 0$. Third row: $0 \le \theta ^D < 1$. Fourth row: $\theta ^D > 1$. Inside each row, the panels are ordered by ascending values of $\eta ^D$ (Color figure online).

To illustrate model fit at the level of respondents, we plotted the aggregated interval responses against 50 posterior draws of their predicted interval responses for 16 randomly selected respondents (Fig. 6). In the plot, the interval responses of a person are aggregated across items by counting how often each of the possible response values is included in the response intervals (e.g., the value .53 might be included in the three intervals $[.50, .54], [.32, .55]\,\,\textrm{and}\,\,[.53, .87]$, leading to a density value of 3). The plot shows the empirical distribution of response values of a respondent as a solid, dark-blue line. In contrast, multiple, randomly-drawn posterior-predicted densities are indicated by light-blue color. Figure 6 reveals that the DDRM had a good fit for respondents with a uni-modal distribution of aggregated interval responses (e.g., Respondent 3 in the first row and third column). In contrast, multi-modal response distributions were not well fitted by the model (e.g., Respondent 6 in the second row and second column). Also, aggregated response distributions that are broadly spread across the whole response scale show a higher level of misfit. For instance, the parameter estimates for Respondent 10 (third row, second column) led to an over-prediction of smaller response intervals in the middle of the response scale. In conclusion, for some respondents, additional latent dimensions might be needed to achieve a better fit of response intervals that are located in different regions of the response scale.

An alternative way to judge a models predictive capabilities is leave-one-out cross-validation (LOO). The basic principle of LOO is to fit a model on a dataset multiple times while holding out one response at a time (Gelman et al., 2014, Chapter 7). The held-out responses are subsequently interpreted as potential future data, which can be used to evaluate the predictive validity of the model. The *loo* package (Vehtari et al., 2017) uses Pareto-smoothed importance sampling as a computationally efficient approximation of LOO. Since only one response in the DDRM ($<0.1\%$) and two responses ($0.1\%$) in the BRM were flagged as either bad or very bad ($\hat{k} > 0.7$; Gabry et al., 2019) by the LOO diagnostics, we assume that the LOO estimates are reliable to facilitate an evaluation of the models. An indicator of predictive performance computed from the LOO estimates is $p_\text {loo}$, defined as the difference between $\text {elpd}_\text {loo}$, that is, the LOO estimate for the expected log pointwise predictive density (with higher values indicating better fit), and the non-cross-validated log posterior predictive density. The $p_\text {loo}$ statistic can be interpreted as the effective number of parameters (Gelman et al., 2014; Vehtari et al., 2017). Essentially, the value of $p_\text {loo}$ should be smaller than the actual number of parameters and the number of responses. For both models, the BRM ($p_\text {loo} = 179.1$, SE $= 10.3$) and the DDRM ($p_\text {loo} = 514.2$, SE $= 11.6$), $p_{loo}$ was smaller than the number of parameters (BRM: $p = 252$, DDRM: $p = 562$) as well as the number of responses (BRM: $n = 2,664$, DDRM: $n = 7,992$). This indicates that both models had a satisfactory predictive performance.

**Fig. 7:**
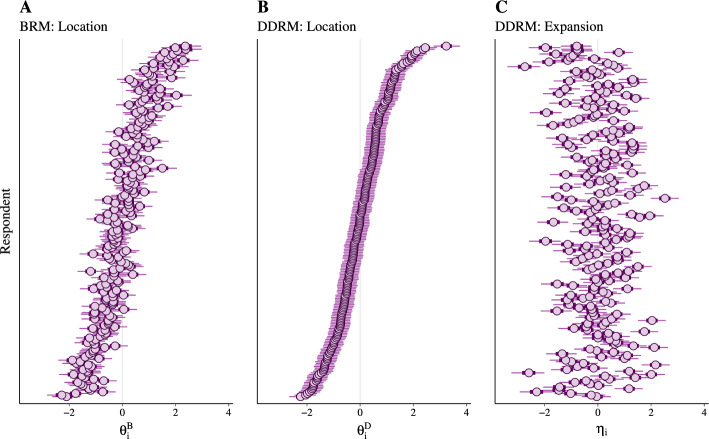
Posterior estimates for the BRM and DDRM person parameters. *Note.* Panel A: Central tendency of Extraversion based on the BRM. Panel B: Central tendency of Extraversion based on the DDRM. Panel C: Variability in Extraversion based on the DDRM. Point estimates show the posterior median whereas dark and light segments show the $50\%$ and $95\%$ equal-tailed posterior intervals, respectively. Across all three panels, individuals are ordered identically depending on their estimate for the DDRM location parameter $\theta ^{D}_i$ (Panel B).

**Fig. 8:**
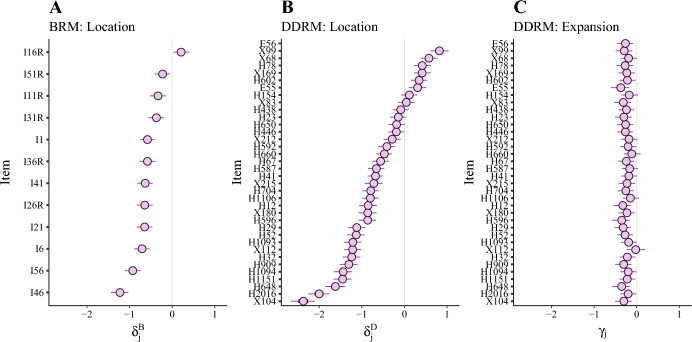
Posterior estimates for the BRM and DDRM item parameters. *Note.* Panel A: Item difficulty for the BRM. Panel B: Item difficulty for the DDRM location dimension. Panel C: Item easiness for the DDRM expansion dimension. Point estimates show the posterior median whereas dark and light segments show the $50\%$ and $95\%$ equal-tailed posterior intervals, respectively. Across all three panels, items are ordered identically depending on their estimate for the DDRM location parameter $\delta ^{D}_j$ (Panel B).

#### Parameter Estimates

Figure 7 shows the estimated person parameters of the BRM (Fig. 7A) and the DDRM (Fig. 7 B and C), which are located on a standard-normal scale due to the standard-normal prior. In all three panels, individuals are ordered by the location estimates of the DDRM (i.e., $\theta _i^D$, Fig. 7B). Comparing the location estimates of the BRM (Fig. 7A) and the DDRM (Fig. 7B), we clearly see a correlation between $\theta ^{B}_i$ and $\theta ^{D}_i$ (correlation estimates are reported in Sect. 5.4.4). On the other hand, $\theta ^{D}_i$ (Fig. 7B) and $\eta _i$ (Fig. 7C) seem to be mostly uncorrelated with a slight curvilinear trend at extreme levels of $\theta ^{D}_i$. Although estimates were more precise for the location parameters $\theta ^{D}_i$ than for the expansion parameters $\eta _i$, the substantial variance of the estimates (relative to the credibility intervals) clearly allows for measuring differences between respondents with respect to all three person parameters. In summary, Fig. 7 illustrates the convergent validity of the BRM and the DDRM with respect to the location dimension, and also the distinction between the location dimension and the expansion dimension within the DDRM.

In the item domain depicted in Fig. 8, the location parameters ($\delta ^{B}_j$, Fig. 8A; $\delta ^{D}_j$, Fig. 8B) and expansion parameters ($\gamma _j$, Fig. 8C) exhibit an overall negative bias compared to the estimates for the person parameters. For the location dimension this could mean that items were overall on the easy side; it might also be an indication of socially desirable answering. For the expansion dimension, this negative bias has no natural interpretation as there is no such thing like a neutral interval width. The estimates for the location dimension $\delta ^{D}_j$ of the DDRM (Fig. 8B) vary across a large range of roughly four standard deviations. In contrast, estimates for the expansion dimension $\gamma _j$ (Fig. 8C) cover only a small range of values compared to the variance of corresponding person parameters $\eta _i$. This mirrors the unbalanced variance ratio of the manifest response interval widths discussed above (see Sect. 5.4.1). In conclusion, the item domain did only have a minor impact on the interval widths, which could be interpreted in two ways. On the one hand, respondents’ variability in Extraversion could be relatively stable across different items, consistent with the findings of Fleeson (2001). On the other hand, the negligible variance of expansion parameters in the item domain could have been caused by respondents’ response styles. The extent to which such response styles occur should be investigated in the future.

Since the BRM concerns one-dimensional data (location) and the DDRM concerns two-dimensional data (location and expansion), a direct comparison of the corresponding item-precision parameters is not meaningful. Nonetheless, within each model, low precision can be used to detect potentially problematic items. In case of the DDRM, this means that respondents answered the respective item in a way that was not consistent with responses given for other items, both regarding interval location and interval width. To give an intuition, we discuss the two items with the lowest precision parameters. The content of these items reveals the potential pitfalls of using an interval-response format. For instance, the item “I am not easily amused” suggests that the use of items that involve more than one semantic direction to reason about (i.e., “not” and “easily”) may be especially problematic when using the RS2 format. Moreover, the item “I love surprise parties” could pose the problem that surprise parties do not happen very frequently, and consequently, respondents might not have had a sufficient number of experiences to assess the variability of their agreement. It is also illustrative to consider the three items with the highest precision parameters: “I cheer people up”, “I feel comfortable around people”, and “I make friends easily.” We can expect respondents to have experienced multiple instances of situations where the described behaviors could have potentially occurred. Overall, this means that the precision parameter is useful for evaluating the alignment of the location and expansion dimension of an item. Precision can only be high if an item allows for a good discrimination in both dimensions. On the flip side, low precision estimates can be used to detect (and possibly remove) inconsistent items.

Lastly, the three scaling parameters were very similar in size across models and dimensions ($\alpha ^{B}$: median $= 0.35$, $95\% \text { HDI} = [0.31,0.39]$; $\alpha ^{D}_{\lambda }$: median $= 0.35$, $95\% \text { HDI} = [0.32,0.38]$; $\alpha ^{D}_{\epsilon }$: median $= 0.38$, $95\% \text { HDI} = [0.34,0.42]$). This is due to the structural similarity of the BRM and the DDRM location dimension, which is further validated in the next section.

#### Convergent Validity of Location Estimates Across Response Formats

Concerning manifest responses, the correlation between the RS2 response-interval locations and the RS1 responses was high ($r =.81$, $95\% \text { CI} =[.76,.85]$), which supports the convergent validity of the RS1 and RS2 response formats. Similar to the raw mean scores, the person parameters $\theta _i$ of the BRM and the DDRM, respectively, were also highly correlated (median $=.87$, $95\% \text { HDI} = [.82,.91]$), supporting the convergent validity of these parameters. The high correlation is especially informative given that the items of the two Extraversion scales differed, and only had an overlap with respect to a subset of facets. Hence, our results provide strong evidence that, for personality questionnaires, the RS2 format can be used in place of the RS1 format to measure the overall strength of agreement or disagreement. Moreover, the use of the IRT models (i.e., both the BRM and the DDRM) considerably increased the degree of convergent validity (roughly 10% additionally explained variance).

#### De-Correlating the Location and Expansion Dimension

The simulation study showed that raw mean scores for the RS2 (i.e., interval locations and widths) are necessarily correlated due to the bounded response scale. In contrast, the DDRM is able to recover the correlation structure of the latent location and expansion parameters, even if the true correlation is zero. To investigate these issues empirically, we computed the correlations of interest for raw mean scores and for the latent DDRM parameters. In the case of manifest responses, again, we computed the correlation between the mean scores for absolute deviance from the scale midpoint ($Y_{\text {ADSM}} = |0.5 - \tfrac{Y_{L} + Y_{U}}{2} |$) and the mean scores for the interval widths, which was $r = -.53$ ($95\%$ CI $= [-.55,-.51]$). While this correlation is large, it is still smaller than the average correlation in the simulation study ($r = -.74$). This difference is probably caused by the relatively large scaling parameter in the simulation study, which pushes the response intervals more towards the bounds of the response scale and thereby exacerbates the scale-inherent correlation described above.

Compared to the correlation of manifest scores, the dependence of the absolute location parameter and the expansion parameter of the DDRM (i.e., $|\varvec{\theta ^{D}}|$ and $\varvec{\eta }$, respectively) was estimated to be less strong with a posterior median of $r = -.18$ ($95\% \text { HDI} = [-.24,-.13]$). Since the simulation showed that the DDRM can even recover zero correlations on the latent scale, the estimated negative correlation provides some evidence for a non-linear relationship of location and expansion. Substantively, this would indicate that respondents generally prefer to provide either smaller intervals at the boundary of the response scale or larger intervals at the center of the response scale, and that this correlation is not merely due to the bounded nature of the scale. Overall, the empirical example thus confirms our findings in the simulation study that using the DDRM substantially reduces the scale-inherent dependence of the manifest mean scores. It therefore helps to identify artifacts caused by the bounded scale that could otherwise mask the true structure of the latent constructs and obstruct the analysis of correlations.

## General Discussion

Our first aim was to develop and evaluate a suitable IRT model for the dual-range slider (RS2) response format in terms of parameter recovery and model fit. The simulation study demonstrated a good recovery of the DDRM’s parameters. However, the precision of the estimated person expansion parameters $\eta _i$ was significantly lower than that of the remaining parameters. This lack of precision on the expansion dimension is also illustrated by the item-information curves for the person parameters and can be explained by the model’s asymmetrical latent parameterization of the Dirichlet distribution (i.e., two tandem parameters working in opposite directions for the location dimension, but only a single parameter for the expansion dimension). For applications with a focus on the expansion or variability dimension (which corresponds to the interval width), one may consider re-parameterizations of the DDRM with higher item information for this dimension in the future.

Regarding model fit in our empirical example application, the results for the DDRM were ambiguous. Model-performance statistics (LOO) were unproblematic while the graphical model checks revealed some misfit. The posterior-predicted distributions for the lower and upper bound of the response interval showed a satisfactory fit, but the DDRM predicted too many narrow intervals in the middle of the response scale. Thus, the model seemed to be lacking flexibility regarding the response-interval widths. However, to our knowledge, there is no competitor model against which our model could have been tested. By developing the DDRM, we proposed a first IRT modeling approach for interval responses, which can be further refined for future applications.

As a second aim, we focused on the convergent validity of the two response formats single-range slider (RS1) and RS2 and the corresponding models. For this purpose, we assessed the correlation of person location parameters estimated by the BRM and the DDRM. This correlation was very high, which provides evidence for the convergent validity of the BRM and DDRM location parameters, and consequently, also of the RS1 and RS2 formats. Hence, the RS2 format may be used in place of the RS1, especially if not only the location dimension but also the expansion dimension is of interest. Thereby, our study contributes to the literature by providing partial evidence for the validity of the interval-response format through direct comparison to a well-established response format (i.e., the visual analogue scale; see Ellerby et al., 2022).

Third, we investigated possible benefits of fitting the DDRM compared to using raw mean scores. Concerning convergent validity, the correlation of location estimates was larger for the latent parameters of the BRM and the DDRM than for the raw mean scores (i.e., the correlation of RS1 responses with RS2 interval locations). Concerning scale-inherent dependencies, the two person parameters of the DDRM for the location and the expansion dimension were less correlated than the corresponding raw mean scores (i.e., interval location and width). This provides evidence for the discriminant validity of the DDRM person estimates on the two dimensions. Thus, we provide a model-based alternative to correction methods that aim at compensating for the detrimental effects of the bounds of a response scale (see Mestdagh et al., 2018, for an example of a correction method for single-response formats). The DDRM might also be useful for improving estimates of the test-retest reliability of interval responses, and thus, to investigate research questions regarding the temporal stability of individual differences in the variability of behaviors and states (Fleeson, 2001).

### Limitations and Future Research

In the present article, we assumed that the interval widths of the RS2 format and, respectively, the expansion dimension of the DDRM represent the variability with respect to the same latent trait measured by the location dimension. A potential problem with this assumption is that respondents might use the RS2 format to describe their subjective uncertainty about the central tendency. In this case, the expansion parameter $\eta _i$ would rather measure respondents’ level of uncertainty instead of the variability of the latent trait across time. Ambiguous interpretations of the task or the item text might further influence how respondents set the width of an response interval. Thus, variability of the trait could be confounded with subjective uncertainty and ambiguity, which might in turn bias model-based inferences about the central tendency and variability of the trait. This is of course an issue that cannot be addressed merely by modeling but rather by further empirical validation studies testing the assumption that the expansion dimension actually measures variability in the latent trait. First, it should be tested whether response intervals and the DDRM parameters are stable across time, both with respect to the location and the expansion dimension. A follow-up study could then combine the RS2 format with experience sampling of the latent trait across a longer time period (Fleeson, 2001). Based on the correlation of the DDRM’s expansion dimension with the individual variance of the behavioral distribution across time, one could test whether the RS2 is actually suitable for measuring variability in behavior.

The RS2 format might also introduce new types of response styles. A plausible and problematic response style concerns the preference for minimum-width intervals because it can potentially occur in combination with extreme interval locations (i.e., extreme response style; see Baumgartner & Steenkamp, 2001, for an overview), but also with intervals that are located in the middle of the scale (i.e., midpoint-response style). In contrast, a response style that is associated with maximum-width intervals will always yield a midpoint-response for the interval location (i.e., midpoint-response style). Such response biases would affect both the location parameter $\theta _i$ as well as the expansion parameter $\eta _i$. A possibility to better handle these extreme responses could be an extension of the DDRM to a zero–one-inflated model (see Molenaar et al., 2022, for examples of model extensions to uni-dimensional models). Even though we only found a low proportion of responses at the boundaries (see Sect. 5.4.1), one could improve model fit by extending the model by a mixture distribution with a certain probability of responses at the boundaries. Moreover, future research should assess discriminant validity of the expansion dimension, namely, that it actually differs from a mere response preference for a certain interval width. This could be done via multidimensional modeling (Wetzel & Carstensen, 2017) of multiple traits (e.g., the big five). In such a model, a strong common factor in the expansion dimension that loads on all items would indicate the presence of an *interval-width response style*. This would mean that the interval width is governed by a respondent’s personal preference for a certain width instead of the different constructs of interest. Given that we fitted the DDRM as a Bayesian model, another direction for future research concerns its implementation in a frequentist framework.

Our empirical example also had some limitations. We used an unbalanced design with a larger number of items for the RS2 format than for the RS1 format. Whereas this is beneficial for model development of the DDRM, which was our foremost intent, the use of different content and number of items means that we could not directly compare responses and item parameters between the BRM and the DDRM. Another limitation concerns the response scales that were displayed to the respondents. These scales showed the exact numerical values above the visual adjustable sliders, which led to response modes for round figures (e.g., 10, 20, 30, etc.). Hence, future studies should avoid showing exact numerical values or anchors for round figures. Furthermore, we did not control for the type of digital device used by the respondents, which might have influenced response behavior.

### Conclusion

We developed a new IRT model for interval responses, the Dirichlet dual response model (DDRM), as an extension to the beta response model (BRM; Noel & Dauvier, 2007), which provides estimates of the central tendency and the variability of a latent trait. We demonstrated the convergent validity of the location dimension both for manifest responses and the latent parameter estimates of the DDRM and the BRM. Moreover, we showed that the estimation of latent parameters reduces the scale-inherent dependence of interval locations and widths. Overall, parameter recovery and model fit of the DDRM were satisfactory while there was some misfit regarding the RS2 interval widths. Also, the latent person parameters for the expansion dimension showed a lower precision of parameter recovery, while the variance in empirical parameter estimates was still sufficient for measuring differences between respondents. Dual range sliders could thus be of great utility for applications where both the central tendency and the variability or uncertainty regarding a latent trait, attitude, or attribute is of primary interest.

## Abbreviations and Parameter Interpretations

- RS1: single-range slider
- RS2: dual-range slider
- IRT: item response theory
- BRM: beta response model
- DDRM: Dirichlet response model
- MCMC / HMC: Markov Chain Monte Carlo / Hamiltonian Monte Carlo
- HDI: highest density interval (for a given posterior distribution; Bayesian)
- CI: confidence interval (frequentist)
- LOO: leave-one-out cross validation
- $\widehat{R}$: Statistic for the diagnosis of MCMC convergence
- ESS: effective sample size
- RMSE: root mean square error
- ADSM: absolute deviance from scale midpoint
- Model Parameters:
  - $\theta _i$: person location (central tendency)
  - $$\delta _j$$: item difficulty
  - $\eta _i$: person expansion (i.e., variability, uncertainty etc.)
  - $$\gamma _j$$: item expansion
  - $$\tau _j$$: item precision
  - $\alpha , \alpha _{\lambda }, \alpha _{\epsilon }$: scaling ($\lambda$: location dimension, $\epsilon$: expansion dimension)
  - Parameter superscripts $\theta ^B$ and $\theta ^D$: Parameter belongs to the BRM or DDRM, respectively

## Log-Likelihood, Derivatives, and Item Information

### Log-Likelihood

The log-likelihood of the DDRM is

B1 $$\begin{aligned} L(\Theta ; \varvec{Y}) =&\displaystyle \sum _{i = 1}^{I} \displaystyle \sum _{j = 1}^{J} \ln \Gamma (a_{ij} + e_{ij} + d_{ij}) \nonumber \\&-\Bigl [ \ln \Gamma (a_{ij}) + \ln \Gamma (e_{ij}) + \ln \Gamma (d_{ij}) \Bigr ] \nonumber \\&+\Bigl [ (a_{ij} - 1) \ln (y_{ij1}) + (e_{ij} - 1) \ln (y_{ij2}) + (d_{ij} - 1) \ln (y_{ij3}) \Bigr ]. \end{aligned}$$

### First Derivatives

In the following, we derive the first partial derivatives of the log-likelihood function for a fixed item *j*. Note that $\psi (x) = \partial \ln \Gamma (x) / \partial x$ is the digamma function. The first partial derivative of the person location parameter $\theta _i$ is obtained via the chain rule of the total derivative,

B2 $$\begin{aligned} \frac{\partial L(\Theta ; \varvec{Y})}{\partial \theta _{i}}&= \frac{\partial L(\Theta ; \varvec{Y})}{\partial a_{ij}} \frac{\text {d} a_{ij}}{\text {d} \theta _{i}} + \frac{\partial L(\Theta ; \varvec{Y})}{\partial d_{ij}} \frac{\text {d} d_{ij}}{\text {d} \theta _{i}} \nonumber \\&= \Bigl [ \psi (a_{ij} + e_{ij} + d_{ij}) - \psi (a_{ij}) + \ln (y_{ij1}) \Bigr ] \alpha _{\lambda } a_{ij} \nonumber \\&+ \quad \; \Bigl [ \psi (a_{ij} + e_{ij} + d_{ij}) - \psi (d_{ij}) + \ln (y_{ij3}) \Bigr ] (- \alpha _{\lambda }) d_{ij}. \end{aligned}$$

The first partial derivative of the person expansion parameter $\eta _i$ is

B3 $$\begin{aligned} \frac{\partial L(\Theta ; \varvec{Y})}{\partial \eta _{i}}&= \frac{\partial L(\Theta ; \varvec{Y})}{\partial e_{ij}} \frac{\text {d} e_{ij}}{\text {d} \eta _{i}} \nonumber \\ {}&= \Bigl [ \psi (a_{ij} + e_{ij} + d_{ij}) - \psi (e_{ij}) + \ln (y_{ij2}) \Bigr ] \alpha _{\epsilon } e_{ij}. \end{aligned}$$

### Second Derivatives and Item Information

In the following, we derive the second partial derivatives of the log-likelihood function. In doing so, $\psi '(x) = \partial \psi (x) / \partial x$ is the trigamma function. The second partial derivative of the person location parameter $\theta _i$ is obtained by another application of the chain rule of the total derivative,

B4 $$\begin{aligned} \frac{\partial ^2 L(\Theta ; \varvec{Y})}{\partial \theta ^2_{i}}&= \left( \frac{\partial L(\Theta ; \varvec{Y})}{\partial \theta _{i}} \Big / \partial a_{ij} \right) \frac{\text {d} a_{ij}}{\text {d} \theta _{i}} + \left( \frac{\partial L(\Theta ; \varvec{Y})}{\partial \theta _{i}} \Big / \partial d_{ij} \right) \frac{\text {d} d_{ij}}{\text {d} \theta _{i}} \nonumber \\&= \Bigl [ (\zeta _a^{(a)} \alpha _\lambda a_{ij} + \zeta _a \alpha _\lambda ) + (- \zeta _d^{(a)} \alpha _\lambda d_{ij})\Bigr ] \alpha _\lambda a_{ij} \nonumber \\&+\quad \; \Bigl [ (\zeta _a^{(d)} \alpha _\lambda a_{ij} ) + (- \zeta _d^{(d)} \alpha _\lambda d_{ij} - \zeta _d \alpha _\lambda ) \Bigr ] (- \alpha _\lambda ) d_{ij} \end{aligned}$$

with

B5 $$\begin{aligned} \zeta _a&= \Bigl [ \psi (a_{ij} + e_{ij} + d_{ij}) - \psi (a_{ij}) + \ln (y_{ij1}) \Bigr ],\nonumber \\ \zeta _d&= \Bigl [ \psi (a_{ij} + e_{ij} + d_{ij}) - \psi (d_{ij}) + \ln (y_{ij3}) \Bigr ], \nonumber \\ \zeta _a^{(a)}&= \frac{\partial \zeta _a}{\partial a_{ij}} = \psi '(a_{ij} + e_{ij} + d_{ij}) - \psi '(a_{ij}), \nonumber \\ \zeta _d^{(d)}&= \frac{\partial \zeta _d}{\partial d_{ij}} = \psi '(a_{ij} + e_{ij} + d_{ij}) - \psi '(d_{ij}), \nonumber \\ \zeta _a^{(d)}&= \frac{\partial \zeta _a}{\partial d_{ij}} = \psi '(a_{ij} + e_{ij} + d_{ij}), \nonumber \\ \zeta _d^{(a)}&= \frac{\partial \zeta _d}{\partial a_{ij}} = \psi '(a_{ij} + e_{ij} + d_{ij}). \end{aligned}$$

Since the second derivative is a linear combination of ln($y_{ij1}$) and ln($y_{ij3}$) (see (B4) and (B5)), the expectation of the second derivative of the joint log-density can be obtained by replacing ln($y_{ij1}$) and ln($y_{ij3}$) with their expected values $\psi (a_{ij}) - \psi (a_{ij} + e_{ij} + d_{ij})$ and $\psi (d_{ij}) - \psi (a_{ij} + e_{ij} + d_{ij})$, respectively. Hence, the item information for $\theta _i$ is

B6 $$\begin{aligned} \mathcal I_{\theta }&= -\mathbb E \Biggl [ \frac{\partial ^2 L(\Theta ; \varvec{Y})}{\partial \theta ^2_{i}} \Biggl ] \nonumber \\ {}&= -\bigl [ (\zeta _a^{(a)} \alpha _\lambda a_{ij}) + (- \zeta _d^{(a)} \alpha _\lambda d_{ij}) \bigr ] \alpha _\lambda a_{ij} \nonumber \\ {}&-\quad \,\, \bigl [ (\zeta _a^{(d)} \alpha _\lambda a_{ij}) + (- \zeta _d^{(d)} \alpha _\lambda d_{ij}) \bigr ] (- \alpha _\lambda ) d_{ij}. \end{aligned}$$

The second partial derivative of the person expansion parameter $\eta _i$ is

B7 $$\begin{aligned} \frac{\partial ^2 L(\Theta ; \varvec{Y})}{\partial \eta ^2_{i}}&= \left( \frac{\partial L(\Theta ; \varvec{Y})}{\partial \eta _{i}} \Big / \partial e_{ij}\right) \frac{\text {d} e_{ij}}{\text {d} \eta _{i}} \nonumber \\&= \Bigl [ \zeta _e^{(e)} \alpha _\epsilon e_{ij} + \zeta _e \alpha _\epsilon \Bigr ] \alpha _\epsilon e_{ij} \nonumber \\ {}&= \zeta _e^{(e)} \alpha _\epsilon ^{2} e^2_{ij} + \zeta _e \alpha _\epsilon ^{2} e_{ij} \end{aligned}$$

with

B8 $$\begin{aligned} \zeta _e&= \Bigl [ \psi (a_{ij} + e_{ij} + d_{ij}) - \psi (e_{ij}) + \ln (y_{ij2}) \Bigr ], \nonumber \\ \zeta _e^{(e)}&= \frac{\partial \zeta _e}{\partial e_{ij}} = \psi '(a_{ij} + e_{ij} + d_{ij}) - \psi '(e_{ij}). \end{aligned}$$

The item information of $\eta _i$ is thus

B9 $$\begin{aligned} \mathcal I_{\eta }&= -\mathbb E \Biggl [ \frac{\partial ^2 L(\Theta ; \varvec{Y})}{\partial ^2 \eta _{i}} \Biggl ] \nonumber \\ {}&= -\mathbb E \bigl [ \zeta _e^{(e)} \alpha _\epsilon ^{2} e^2_{ij} + \zeta _e \alpha _\epsilon ^{2} e_{ij} \bigr ] \nonumber \\ {}&= -\bigl [\zeta _e^{(e)} \alpha _\epsilon ^{2} e^2_{ij} + \mathbb E(\zeta _e) \alpha _\epsilon ^{2} e_{ij}\bigr ] \nonumber \\ {}&= -(\zeta _e^{(e)} \alpha _\epsilon ^{2} e^2_{ij}). \end{aligned}$$

The cross partial derivative for the person location parameter $\theta _i$ and the person expansion parameter $\eta _i$ is

B10 $$\begin{aligned} \frac{\partial ^2 L(\Theta ; \varvec{Y})}{\partial \eta _{i} \partial \theta _i}&= \frac{\partial }{\partial \eta _{j}} \frac{\partial L(\Theta ; \varvec{Y})}{\partial \theta _{i}} = \left( \frac{\partial L(\Theta ; \varvec{Y})}{\partial \theta _{i}} \Big / \partial e_{ij}\right) \frac{\text {d} e_{ij}}{\text {d} \eta _{i}} \nonumber \\ {}&= \Bigl [ \bigl [ \psi '(a_{ij} + e_{ij} + d_{ij}) \alpha _\lambda a_{ij}) \bigr ] + \bigl [ \psi '(a_{ij} + e_{ij} + d_{ij}) (-\alpha _\lambda ) d_{ij} \bigr ] \Bigr ]\alpha _\epsilon e_{ij}. \end{aligned}$$

The corresponding Fisher information is

B11 $$\begin{aligned} \mathcal I_{\eta \theta }&= -\mathbb E \Biggl [ \frac{\partial ^2 L(\Theta ; \varvec{Y})}{\partial \eta _{i} \partial \theta _i} \Biggl ] \nonumber \\ {}&= - \bigl [ \psi '(a_{ij} + e_{ij} + d_{ij}) (\alpha _\lambda a_{ij} - \alpha _\lambda d_{ij}) \bigr ] \alpha _\epsilon e_{ij} \nonumber \\ {}&= - \psi '(a_{ij} + e_{ij} + d_{ij}) \alpha _\epsilon \alpha _\lambda e_{ij} ( a_{ij} - d_{ij}). \end{aligned}$$

## Footnotes

1 Due to the transformation, the minimum and maximum of the values used in the analysis are $\tfrac{1}{102}$ and $1 - \tfrac{1}{102}$, respectively, which is an arbitrary choice based on the resolution of the original scale. The theoretical endpoints of the scale (i.e., zero and one) cannot be selected by the respondents (this corresponds to the open-response situation described in Samejima, 1973).

2 The original model by Noel and Dauvier (2007) fixes $\alpha$ to 1 and divides everything inside the two exponential functions by 2.

3 This becomes evident when parameterizing the beta distribution in terms of the mean $\mu _{ij} = \tfrac{m_{ij}}{m_{ij} + n_{ij}}, (0< \mu _{ij} < 1)$ and the sample size $\nu _{ij} = m_{ij} + n_{ij} > 0$ where $Var(x_{ij}) = \tfrac{\mu _{ij}(1 - \mu _{ij})}{1 + \nu _{ij}}$. If we increase $m_{ij}$ and $n_{ij}$ such that $\mu _{ij}$ stays constant, the numerator of the variance equation stays constant, but the denominator increases.

4 Graphical illustrations can be found at the OSF repository: https://osf.io/br8fa/. The 95% HDI of the prior distribution $\mathcal {N}(0,1.5)$ is $[-2.94, 2.94]$ and that of the prior distribution $\Gamma (1.5,1.5)$, parameterized in terms of shape and rate, is [0.00, 2.61].

5 A list of the used items can be found at the OSF repository: https://osf.io/br8fa/.

6 The selection criterion for items was an angle with the factor axis of $\alpha \le 30^\circ$ (Reckase, 2009, p. 117).

7 The 95% HDI of the prior distribution $$\text {t}(3,0,2)$$ is $$[-6.36, 6.36] $$ and that of the prior distribution $$\text {t}(3,0,2)$$ truncated to $$(0, \infty )$$ is [0.00, 6.36].

8 The data, R script, and Stan code for fitting the joint model can be found at the OSF repository: https://osf.io/br8fa/. Fitting took approximately 75 min on an i9-9820X processor. The estimation of a simple DDRM model for 200 respondents on 30 items with 4 chains, 500 burn-in iterations, and 500 sampling iterations required approximately 7.2 min. A template for fitting the model can also be found at the above-mentioned OSF repository.

9 The tuning parameter $\text {adapt}_{\Delta }$ was set to.80. We also used random starting values from the interval $[-.2,.2]$.

## Declarations

### Data Availability

The data and analysis scripts for this article are available through the Open Science Framework (OSF): https://osf.io/br8fa/.
